# Supplementary material for: Severe Acute Respiratory Syndrome Coronavirus 2 (SARS-CoV-2) Spike Protein S1 Induces Methylglyoxal-Derived Hydroimidazolone/Receptor for Advanced Glycation End Products (MG-H1/RAGE) Activation to Promote Inflammation in Human Bronchial BEAS-2B Cells
Source: Int J Mol Sci. 2023 Oct 3;24(19):14868. doi: 10.3390/ijms241914868 (PMC10573269; doi:10.3390/ijms241914868)

Supplementary Material: SARS-CoV-2 Spike Protein S1 Induces MG-H1/RAGE Activation to Promote Inflammation in Human Bronchial BEAS-2B Cells

Dominga Manfredelli, Marilena Pariano, Claudio Costantini, Alessandro Graziani, Silvia Bozza, Luigina Romani, Paolo Puccetti, Vincenzo Nicola Talesa and Cinzia Antognelli

Figure S1. Cell morphology, by means of phase-contrast microscopy, of BEAS-2B and A549 cells exposed to 25 and 100 ng/ml SARS-CoV-2 spike protein S1 for 6 and 24 hours and controls (0 ng/ml). Scale bar = 200 μm (BEAS-2B-related images), = 400 μm (A549-related images).

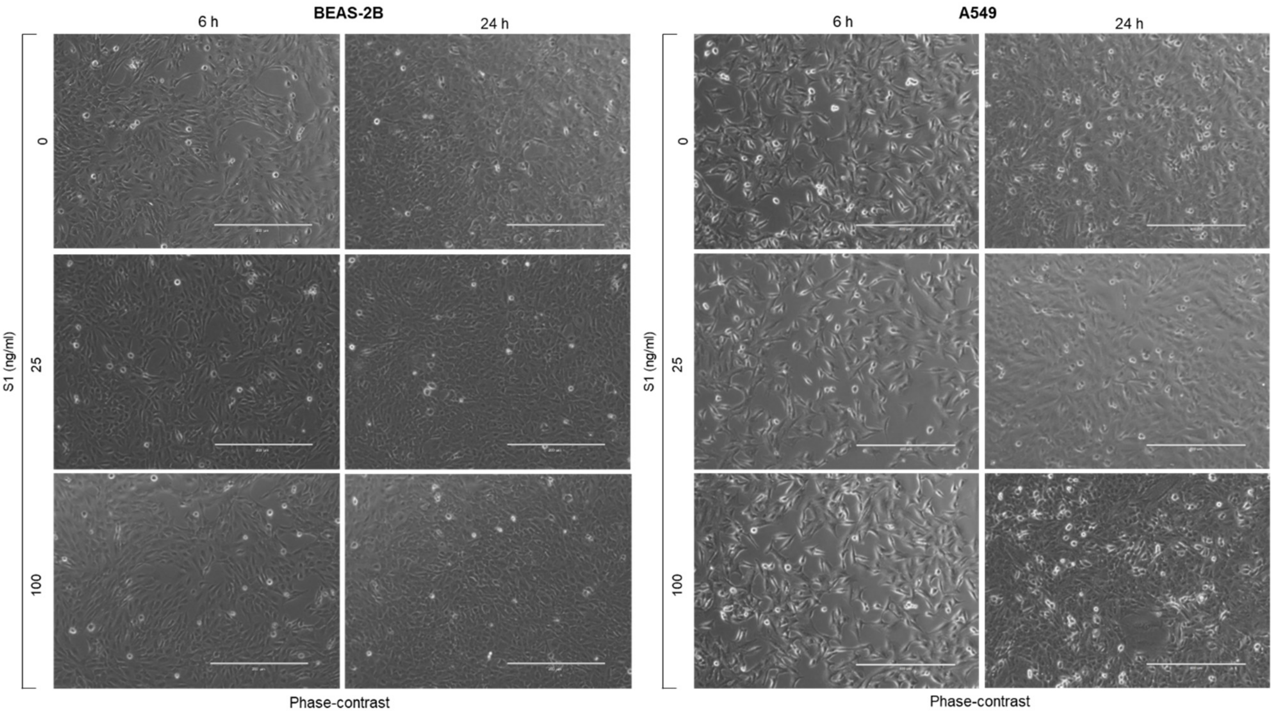

Figure S2. SARS-CoV-2 S1 Spike protein induces inflammatory cytokines in human bronchial BEAS-2B and alveolar A549 epithelial cells. BEAS-2B and A549 cells were stimulated with S1 at a concentration of 100 ng/ml. Six and 24 h post-stimulation, the expression of IL-1β was evaluated by real-time RT-PCR, while TNF-α, IL-6 and IL-8 levels, by ELISA. Data represent mean ± SD (n = 3). *p < 0.05, **p < 0.01, ***p < 0.001.

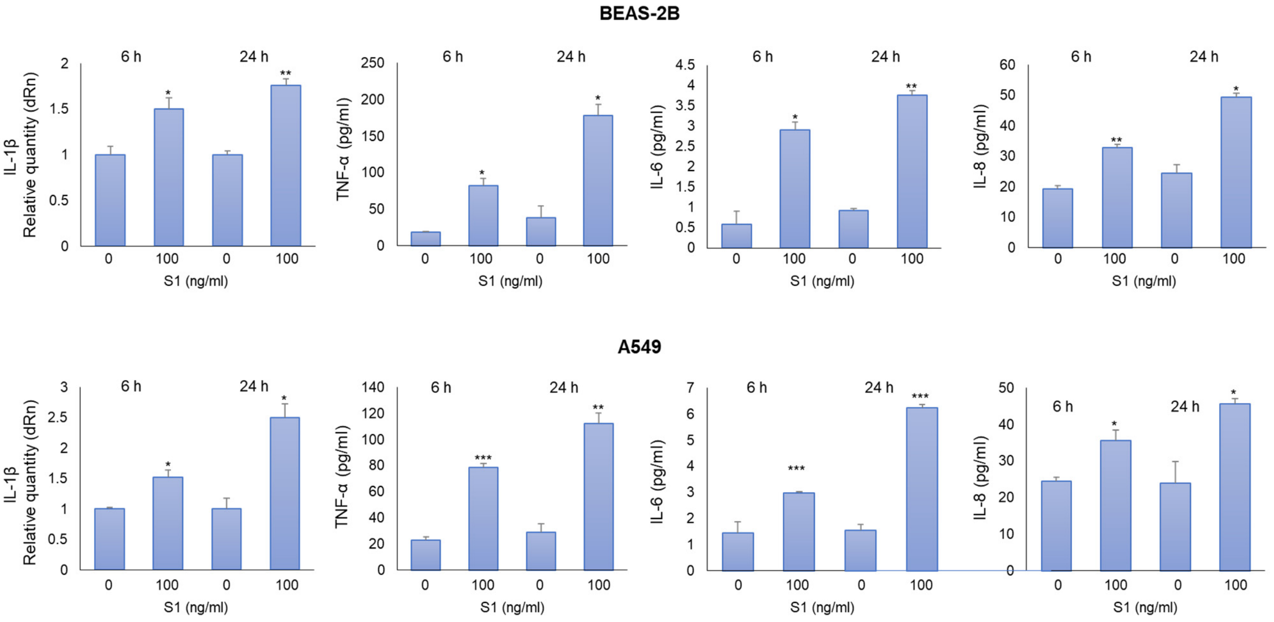

Figure S3. Effect of 10 μM Nrf2 activator (Nrf-A) pre-treatment on ACE2 mRNA expression in BEAS-2B cells. ACE2 expression was evaluated by real-time RT-PCR. (Ct range: 35-38). Data represent mean ± SD (n = 3). Human ACE2 sense (5′- GGAGTGATAGTGGTTGGCATTGTC-3′) and antisense (5′-GCTAATATCGATGGAGGCATAAGGA- 3′) primers. GAPDH primers are reported in the main text.

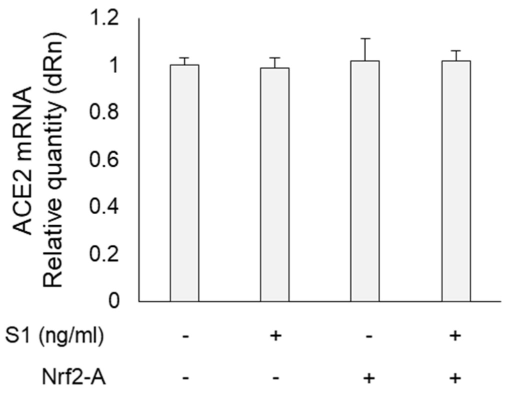

Figure S4. Glyoxalase 1 (Glo1) mRNA expression in alveolar A549 epithelial cells exposed to 25 ng/ml SARS-CoV-2 S1 spike protein for 24 h. Glo1 expression was evaluated by real-time RT-PCR. Data represent mean ± SD (n = 3). *p < 0.05.

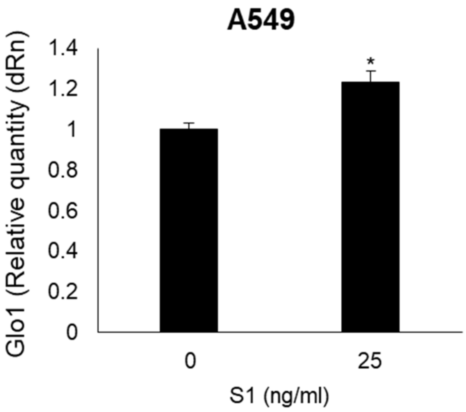

Figure S5. Whole blots reported in Figure 3b, Figure 3d and Figure 5a.

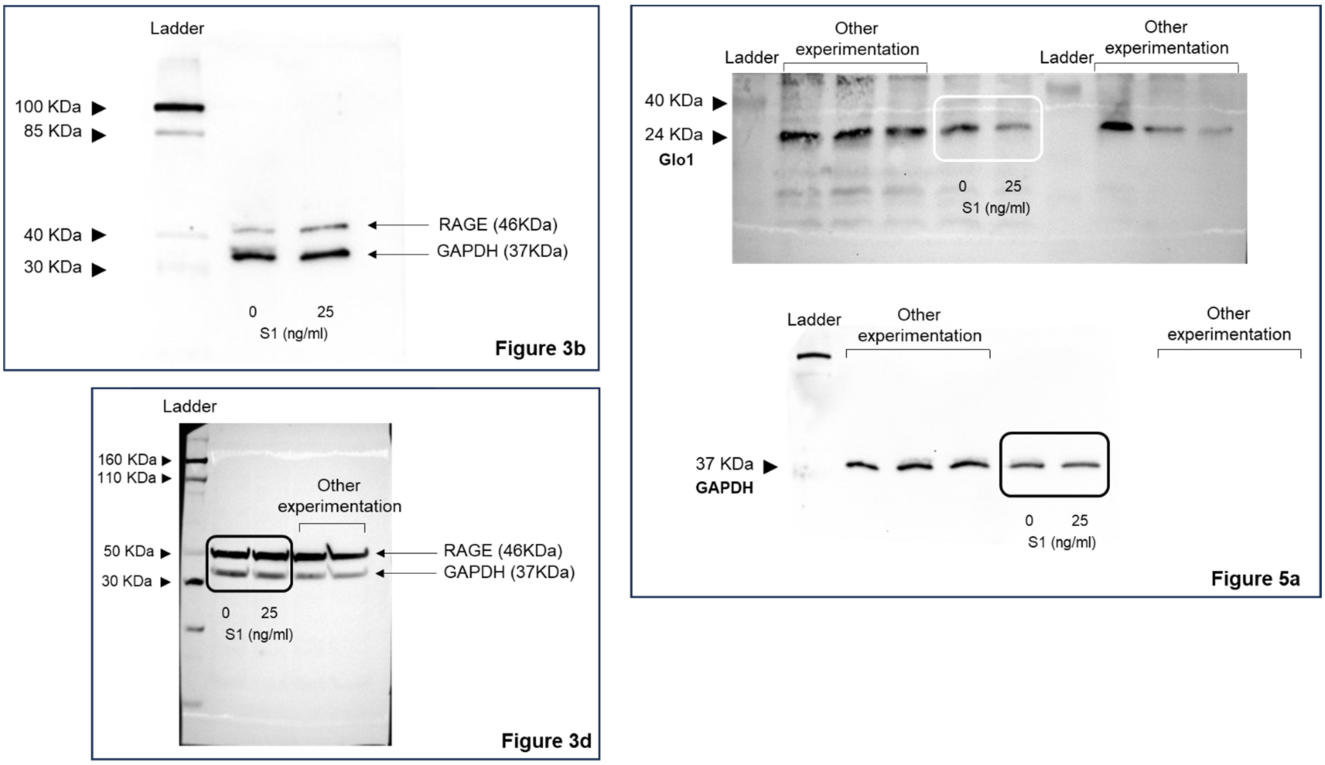

Supplement: Supplementary file 1 [file ijms-24-14868-s001.zip › ijms-2607374-supplementary.pdf]
